# Selection Between Liver Resection Versus Transarterial Chemoembolization in Hepatocellular Carcinoma: A Multicenter Study

**DOI:** 10.14309/ctg.0000000000000070

**Published:** 2019-08-01

**Authors:** Sirui Fu, Jingwei Wei, Jie Zhang, Di Dong, Jiangdian Song, Yong Li, Chongyang Duan, Shuaitong Zhang, Xiaoqun Li, Dongsheng Gu, Xudong Chen, Xiaohan Hao, Xiaofeng He, Jianfeng Yan, Zhenyu Liu, Jie Tian, Ligong Lu

**Affiliations:** 1 Zhuhai Interventional Medical Center, Zhuhai Precision Medical Center, Zhuhai People's Hospital, Zhuhai Hospital of Jinan University, Zhuhai, China;; 2 Key Laboratory of Molecular Imaging, Institute of Automation, Chinese Academy of Sciences, Beijing, China;; 3 University of Chinese Academy of Sciences, Beijing, China;; 4 Department of Radiology, Zhuhai People's Hospital, Zhuhai Hospital of Jinan University, Zhuhai, China;; 5 Department of Biostatistics, School of Public Health, Southern Medical University, Guangzhou, China;; 6 Department of Interventional Treatment, Zhongshan City People's Hospital, Zhongshan, China;; 7 Department of Radiology, Shenzhen People's Hospital, Shenzhen, China;; 8 Interventional Diagnosis and Treatment Department, Nanfang Hospital, Southern Medical University, Guangzhou, China;; 9 Department of Radiology, Yangjiang People's hospital, Yangjiang, China;; 10 Beijing Advanced Innovation Center for Big Data-Based Precision Medicine, Beihang University, Beijing.

## Abstract

Models should be developed to assist choice between liver resection (LR) and transarterial chemoembolization (TACE) for hepatocellular carcinoma.

**METHODS:**

After separating 520 cases from 5 hospitals into training (n = 302) and validation (n = 218) data sets, we weighted the cases to control baseline difference and ensured the causal effect between treatments (LR and TACE) and estimated progression-free survival (PFS) difference. A noninvasive PFS model was constructed with clinical factors, radiological characteristics, and radiomic features. We compared our model with other 4 state-of-the-art models. Finally, patients were classified into subgroups with and without significant PFS difference between treatments.

**RESULTS:**

Our model included treatments, age, sex, modified Barcelona Clinic Liver Cancer stage, fusion lesions, hepatocellular carcinoma capsule, and 3 radiomic features, with good discrimination and calibrations (area under the curve for 3-year PFS was 0.80 in the training data set and 0.75 in the validation data set; similar results were achieved in 1- and 2-year PFS). The model had better accuracy than the other 4 models. A nomogram was built, with different scores assigned for LR and TACE. Separated by the threshold of score difference between treatments, for some patients, LR provided longer PFS and might be the better option (training: hazard ratio [HR] = 0.50, *P* = 0.014; validation: HR = 0.52, *P* = 0.026); in the others, LR provided similar PFS with TACE (training: HR = 0.84, *P* = 0.388; validation: HR = 1.14, *P* = 0.614). TACE may be better because it was less invasive.

**DISCUSSION:**

We propose an individualized model predicting PFS difference between LR and TACE to assist in the optimal treatment choice.

## INTRODUCTION

Worldwide, liver cancer ranks high in terms of incidence and mortality; 70%–90% of these cases are hepatocellular carcinoma (HCC) (1). HCCs are often accompanied by underlying liver dysfunction; therefore, death is caused not only by tumor burden but also by deterioration of liver function (2), which makes it important to avoid unnecessary trauma. For HCC, liver resection (LR) is curative but is highly traumatic, whereas transarterial chemoembolization (TACE) is minimally invasive but may leave some residual tumor. With the advancement of technology, their adaptation has expanded and overlapped (3–8). The definition of “unresectable HCC” is somewhat ambiguous in the guidelines of the European Association for the Study of the Liver (9) and the American Association for the Study of Liver Diseases (10). The latest National Comprehensive Cancer Network guideline states that the performance of LR (in some previous TACE-treated cohorts) is “controversial” (11). Researchers suggest that we should move from an approach of “what can be done” to a “what is worth doing” process (12,13). Thus, selecting treatments for patients based on predecided parameters may be rational (3,14). The existing methods for treatment selection have some limitations, such as subjective outcomes and a lack of validation. A new model that can help making the treatment choice between LR and TACE is required. Furthermore, the model should use proper method to ensure that the survival difference between LR and TACE is causal effect (15). To this end, individualized models to assistant choice between LR and TACE are needed.

During HCC management, biopsy is not a necessity (9,10). Hence, the choice between LR and TACE would be better assisted by noninvasive methods. To collect information for the noninvasive methods, in addition to traditional clinical factors and radiological characteristics (16), radiomics may also be helpful. Radiomics can quantitatively mine data from medical images, including but not limited to size/shape, heterogeneity/texture, and relationships with surrounding tissues. Radiomics can improve diagnostic and prognostic accuracy (17) and is valuable for decision making in cancer (18,19) and liver disease (20).

Therefore, we conducted this multicenter study on patients with HCC, aiming to establish noninvasive individualized models by combining clinical factors, radiological characteristics, and radiomic features. Assisted by the model, we planned to predict prognostic difference associated with LR and TACE and then classified the population for the optimal treatment accordingly.

## METHODS

### Patients

HCC cases from Nanfang Hospital, Shenzhen People's Hospital, Yangjiang People's Hospital, Zhongshan City People's Hospital, and Zhuhai People's Hospital in China were reviewed. After screening the electronic medical record system from 2008 to 2016, we identified 520 HCC cases for which computed tomography (CT) records at diagnosis were available. Inclusion criteria were as follows: (i) HCC confirmed pathologically or clinically; (ii) patients initially treated by LR or TACE; and (iii) at least one radiological disease progression (PD) confirmed by CT/MRI or a follow-up of more than 1 year before the end date. Exclusion criteria were as follows: (i) initially treated by ablation or other treatments; (ii) irregular follow-up; (iii) administration of other combination therapies before PD (such as ablation or systemic therapy); (iv) incomplete initial treatment of intrahepatic lesion (such as positive margin in LR and untreated lesion in TACE); and (v) severe medical comorbidities such as extensive cardiovascular disease (see Figure S1, Supplemental Digital Content 3, http://links.lww.com/CTG/A86).

The 2018 American Association for the Study of Liver Diseases guideline recommended the modified Barcelona Clinic Liver Cancer (BCLC) staging system rather than the TNM system (10). Single HCC > 5 cm was not an optimal candidate for LR and appeared to have a worse prognosis than HCC ≤ 5 cm (10), which resulted in the adoption of stage AB in research and discussed in the European Association for the Study of the Liver guideline (3,9). Thus, we tested whether the application of modified BCLC and stage AB would improve the performance of our model.

The study protocols were approved by the Ethics Committee of Zhuhai People's Hospital. The requirement for informed consent to use the patients' data for medical researches was waived because the data were collected retrospectively. All patient records and information were anonymized and deidentified before analysis.

### Follow-ups and treatments

For the included patients, the follow-up was scheduled every 4–6 weeks before PD or at least for 1 year (without PD), after that, the interval might be increased from 3 to 6 months. Every follow-up included chest radiograms, abdominal CT or MRI, and other necessary laboratory tests. Additional CT or MRI scans were obtained if extrahepatic metastasis was suspected.

The initial treatment between LR and TACE was decided based on the same criteria across hospitals, according to tumor characteristics referring to guidelines (9–11), liver functional status, and the patients' choice. LR was performed with the aim of removing all intrahepatic macroscopic tumors with a margin confirmed by pathological examination. Conventional TACE was performed using the standard method as selectively as possible (9–11). Before PD, no additional LR, TACE, or ablation procedures were performed. Inspired by a previous study (3), for BCLC stage A, some patients with early-stage HCC preferred TACE over LR because TACE was less invasive. For BCLC stage C, patients with suspicious extrahepatic nodule proven to be metastases during the follow-ups, and patients with macrovascular invasion at diagnosis were not excluded because of (i) the limited efficacy of targeted therapies recommended by guidelines (21); (ii) the potential benefit derived from LR and TACE for these patients (3,5,6,8,11,22–24) and treatments should not be delayed (especially for those with macrovascular invasion in portal vein branches or suspicious regional lymph node metastasis). After informing patients of the potential consequences and obtaining informed consent for LR or TACE procedures, the physicians performed LR/TACE as the initial treatment.

### Outcomes

Based on ethical considerations, after PD, to more effectively control the disease, patients could accept other therapies, even those beyond first-line treatments recommended by guidelines (25); however, this might introduce bias in the analysis of overall survival. Therefore, we used progression-free survival (PFS) as the end point (26). PFS was defined as the time from diagnosis to PD or death. PD was defined according to the modified Response Evaluation Criteria in Solid Tumors criteria (27), referring to both target lesion and nontarget lesions (such as macrovascular invasion or extrahepatic metastases).

### Candidate clinical factors and radiological characteristics

**Figure 1.**
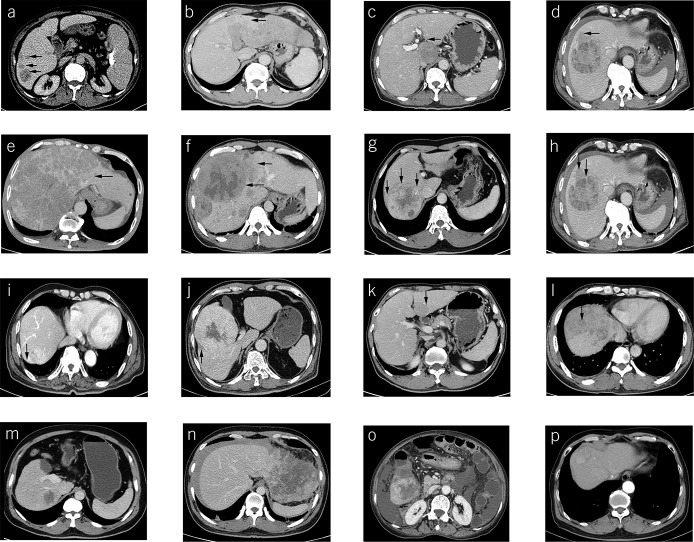
Radiological characteristics of HCC. Arrows show the following: fusion lesions, classified as absent (**a**) or present (**b**); invasive shape, classified as noninvasive (**c**) or invasive (**d**); HCC capsule, classified as absent (**e**), unintegral (**f**), or integral (**g**); HCC capsule breakthrough (**h**); corona enhancement (**i**); corona with low attenuation (**j**); mosaic architecture (**k**); nodule-in-nodule architecture (**l**); and HCC with enhancement, classified as <25% (**m**), 25–50% (**n**), 50–75% (**o**), or >75% (**p**). HCC, hepatocellular carcinoma.

Besides the abovementioned baseline factors and radiomic features, we also included several other factors during model construction: (i) neutrophil-to-lymphocyte ratio; (ii) HCC location: lobe (classified as left, right, and cross); surface (whether close to the liver capsule or not, classified as negative or positive); and (iii) radiological characteristics: fusion lesions, boundary fusion of >2 lesions, classified as absent (Figure 1a) or present (Figure 1b); invasive shape, protrusions like crab foot, classified as noninvasive (Figure 1c) or invasive (Figure 1d); HCC capsule, classified as absent (Figure 1e), unintegral (Figure 1f), or integral (Figure 1g); HCC capsule breakthrough (Figure 1h), HCC growth beyond a preexisting capsule, classified as absent or present; corona enhancement (16) (Figure 1i), a transient zone or rim of enhancement around HCC, classified as absent or present; corona with low attenuation (Figure 1j), classified as absent or present; mosaic architecture (16) (Figure 1k), a mass of randomly distributed internal nodules or compartments differing in enhancement, classified as absent or present; nodule-in-nodule architecture (16) (Figure 1l), the presence of a nodule within a larger nodule or mass, classified as absent or present; and HCC with enhancement, classified as <25% (Figure 1m), 25%–50% (Figure 1n), 50%–75% (Figure 1o), or >75% (Figure 1p).

### Radiomic feature extraction

Baseline CT images were obtained using the scanners in the collaborative hospitals (see Table S1, Supplemental Digital Content 2, http://links.lww.com/CTG/A85). All CT images were retrieved from the picture archiving and communication system.

Because HCC capsule was identified in the portal phase rather than the arterial phase (16), which could increase the accuracy of lesion segmentation, the portal phase was used for feature extraction. The target lesion was selected according to the modified Response Evaluation Criteria in Solid Tumor (27) (see Text S1, Supplemental Digital Content 1, http://links.lww.com/CTG/A84). In total, we extracted 708 radiomic features (see Text S2, Supplemental Digital Content 1, http://links.lww.com/CTG/A84). After randomly sampling 20% cases per hospital, we used morphologic perturbations to test the robustness and redundancy (see Text S3, Supplemental Digital Content 1, http://links.lww.com/CTG/A84) (28). The related program, instruction, and example of patients can be downloaded from http://www.radiomics.net.cn/post/108.

### Statistical analysis

Continuous variables were expressed as mean (SD) or median (range) based on whether they were normally distributed or not, and groups were compared using the *t*-test or Wilcoxon rank-sum test as appropriate. Categorical variables were expressed as percentages, and groups were compared using the Pearson χ^2^ test or Fisher exact test.

**Figure 2.**
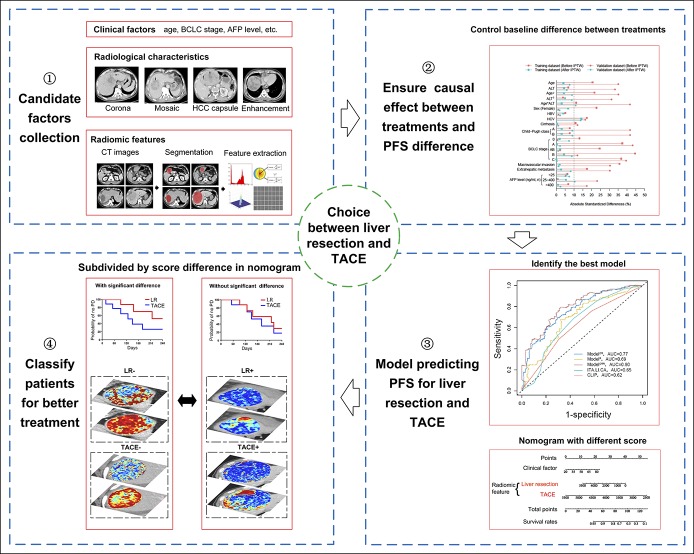
Design of the study. AFP, alpha-fetoprotein; BCLC, Barcelona Clinic Liver Cancer; CT, computed tomography; LR, liver resection; PFS, progression-free survival; TACE, transarterial chemoembolization.

For model construction, after randomly splitting the patients into the training and validation data sets, we used the following steps to develop our individualized model. First, we collected the clinical factors, radiological characteristics, and radiomic features for our model. Second, inverse probability of treatment weighting (IPTW) based on the propensity score was used to weight the patients in the training and validation data sets separately (see Text S4, Supplemental Digital Content 1, http://links.lww.com/CTG/A84) (29,30), because our model aimed to ensure the causal effect (31) in treatment outcome differences between LR and TACE. Third, the Cox proportional hazard model was applied to predict the PFS associated with LR and TACE using the weighted training data set. The candidate factors and their interactions with treatments were selected by the Least Absolute Shrinkage and Selection Operator method (32) and the backward stepwise selection method using the Akaike information criterion. We constructed 3 models to evaluate whether the combination of clinical factors, radiological characteristics, and radiomic features was necessary: Model^CR^ included clinical factors (C) and radiological characteristics (R); Model^R^, radiomic features (R); and Model^CRR^, clinical factors (C), radiological characteristics (R), and radiomic features (R). We also compared our developed models with 2 other existing models (ITA.LI.CA (33) and CLIP (34)) reported to be superior. The predictive accuracy of the developed models was assessed by both discriminations measured by the time-dependent receiver operating characteristic curve for 1-, 2-, and 3-year PFS and Harrell concordance index (C-index), and calibration evaluated by the calibration plot. A nomogram of the best developed model was obtained. Fourth, we calculated the score difference between LR and TACE of the best model; the cutoff value of the score difference was used to subdivide the patients for classification. We used Kaplan-Meier plots to compare the difference between the subgroups. The abovementioned steps are illustrated in Figure 2.

All statistical tests performed were two sided, and *P* values <0.05 were considered statistically significant. We analyzed data by the R statistical package (http://www.r-project.org/).

## RESULTS

### Characteristics of the study population

Among the 520 patients, the training and validation data sets had 302 and 218 patients, respectively (Table 1). The patients initially treated by LR and TACE were 97 (32%) and 205 (68%) in the training data set and 65 (30%) and 153 (70%) in the validation data set, respectively. During follow-up, 338 patients (training: 193; validation: 145) showed PD, and 182 patients (training: 102; validation: 80) died. Baseline differences between LR and TACE groups before IPTW were shown in Table S2 (see Supplemental Digital Content 2, http://links.lww.com/CTG/A85).

**Table 1.**
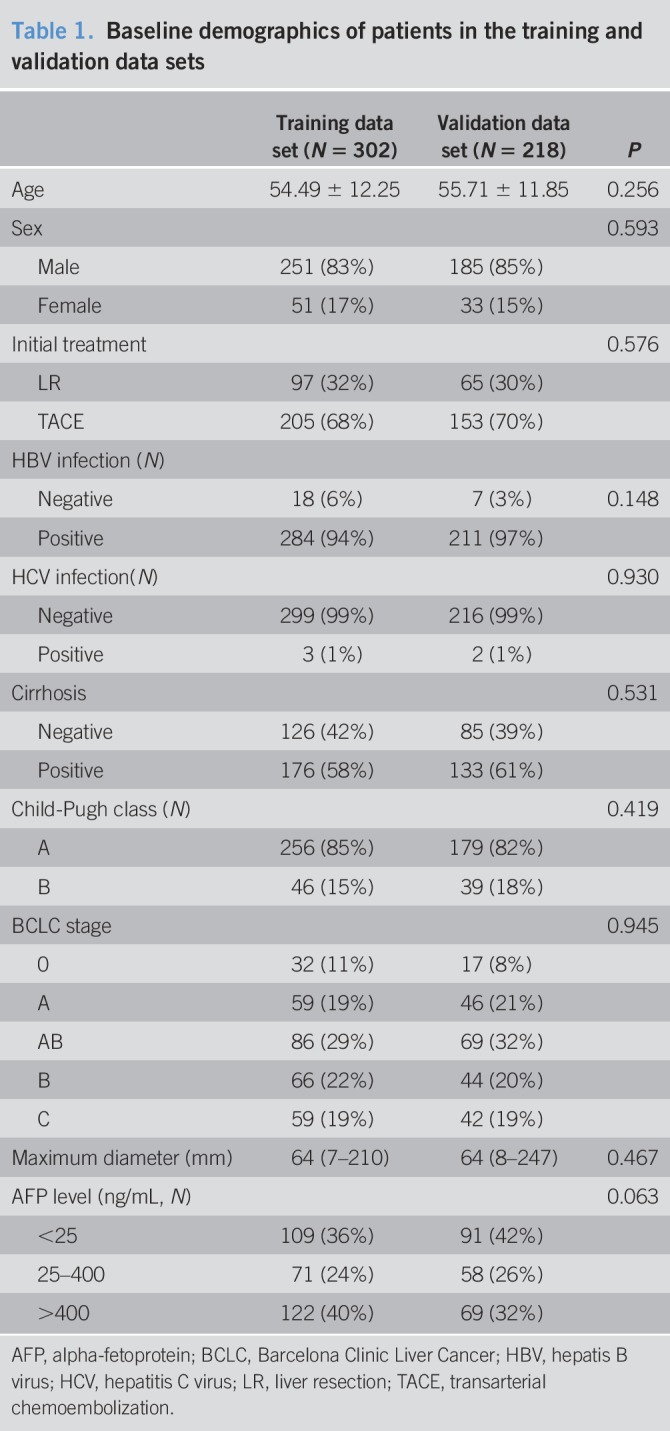
Baseline demographics of patients in the training and validation data sets

### Radiomic features and IPTW results

For the 708 radiomic features, 607 features with intraclass correlation coefficient > 0.75 were used for the final analysis (see Figure S2, Supplemental Digital Content 3, http://links.lww.com/CTG/A86). IPTW controlled the baseline differences between LR and TACE groups such as tumor burden, liver function, and chronic liver disease (see Text S5, Supplemental Digital Content 1, http://links.lww.com/CTG/A84) in both data sets (see Figure S3a,b, Supplemental Digital Content 3, http://links.lww.com/CTG/A86) and most standardized differences <10% (see Table S2, Supplemental Digital Content 2, http://links.lww.com/CTG/A85). After IPTW, there were 512 patients included for PFS analysis (training: 298 patients; validation: 214 patients).

### Model development and validation

**Figure 3.**
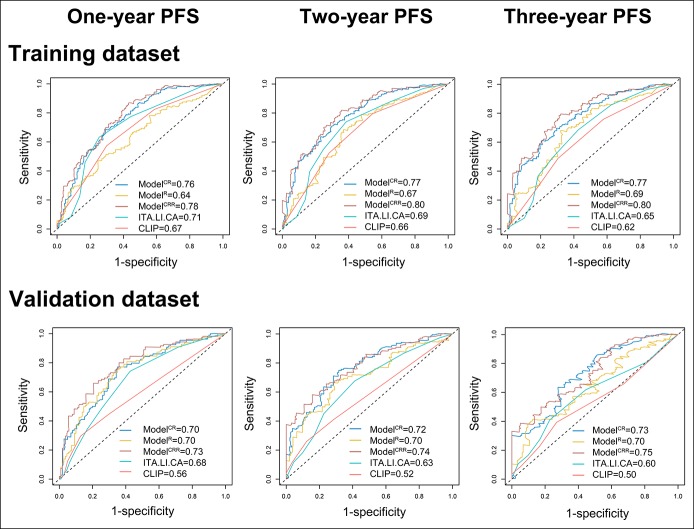
Testing the prognostic accuracy of the constructed models. For 1-, 2- and 3-year progression-free survival, compared with Model^CR^, Model^R^, ITA.LI.CA, and CLIP, Model^CRR^ containing clinical factors, radiological characteristics and radiomic features show the best area under the curve, both in the training and validation data sets. Model^CR^ included clinical factors (C) and radiological characteristics (R); Model^R^ included radiomic features (R); Model^CRR^ included clinical factors (C), radiological characteristics (R), and radiomic features (R). PFS, progression-free survival.

**Figure 4.**
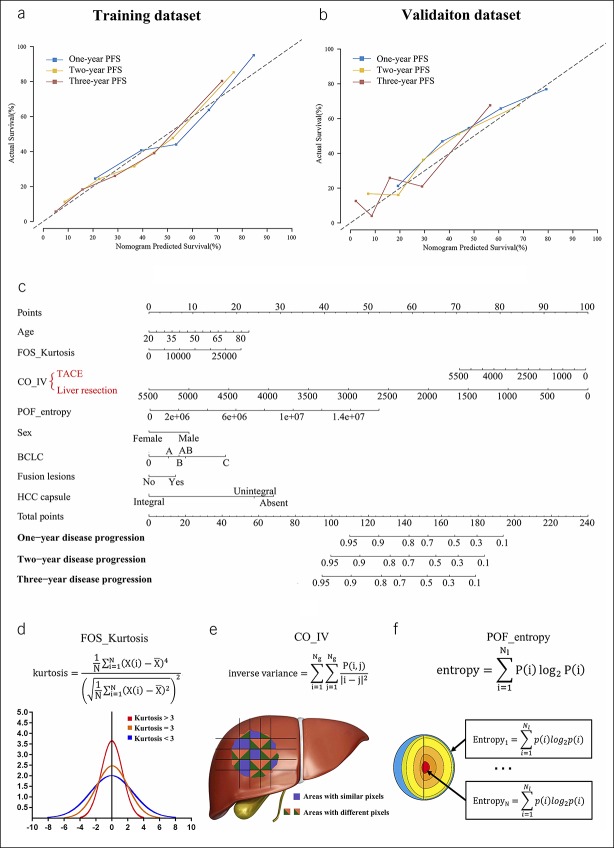
Calibration, nomogram, and schematic diagram of identified radiomic feature. The nomogram shows good calibration for the 1-, 2-, and 3-year PFS, both in the training (**a**) and validation (**b**) data sets. We construct a nomogram of Model^CRR^ (**c**). The calculation formulas and schematic diagram of the 3 radiomic features are displayed (**d**, **e**, and **f**). Model^CRR^ included clinical factors (C), radiological characteristics (R), and radiomic features (R). BCLC, Barcelona Clinic Liver Cancer; HCC, hepatocellular carcinoma; PFS, progression-free survival; TACE, transarterial chemoembolization.

Among the 5 models, Model^CRR^, containing treatments, age, sex, BCLC stage, fusion lesions, HCC capsule, and 3 radiomic features (FOS_Kurtosis, CO_IV, and POF_entropy), showed the best area under the curve for 1-, 2- and 3-year PFS (training: 0.78, 0.80, and 0.80; validation: 0.73, 0.74, and 0.75, respectively, Figure 3). The differences between Model^CRR^ and the other 4 models were statistically significant (see Table S3, Supplemental Digital Content 2, http://links.lww.com/CTG/A85), with Model^CRR^ having the highest Harrell C-index (Model^CR^: 0.696; Model^R^: 0.626; Model^CRR^: 0.707; ITA.LI.CA: 0.650; CLIP: 0.620). Model^CRR^ showed good calibration in both data sets (Figure 4a,b). In addition, Model^CRR^ showed an improved performance than the model without modified BCLC and stage AB (see Text S6, Supplemental Digital Content 1, http://links.lww.com/CTG/A84, and see Figure S4, Figure S5, Supplemental Digital Content 3, http://links.lww.com/CTG/A86).

Based on the above results, a nomogram of Model^CRR^ was constructed (Figure 4c). In the nomogram, 3 radiomic features were included, with their calculation formulas and schematic diagram displayed (Figure 4d–f) and a detailed explanation provided in the discussion. CO_IV was identified to have an interaction with treatments, and therefore, different scores were assigned for CO_IV, facilitating the predicted PFS for LR and TACE each.

### Population classification

**Figure 5.**
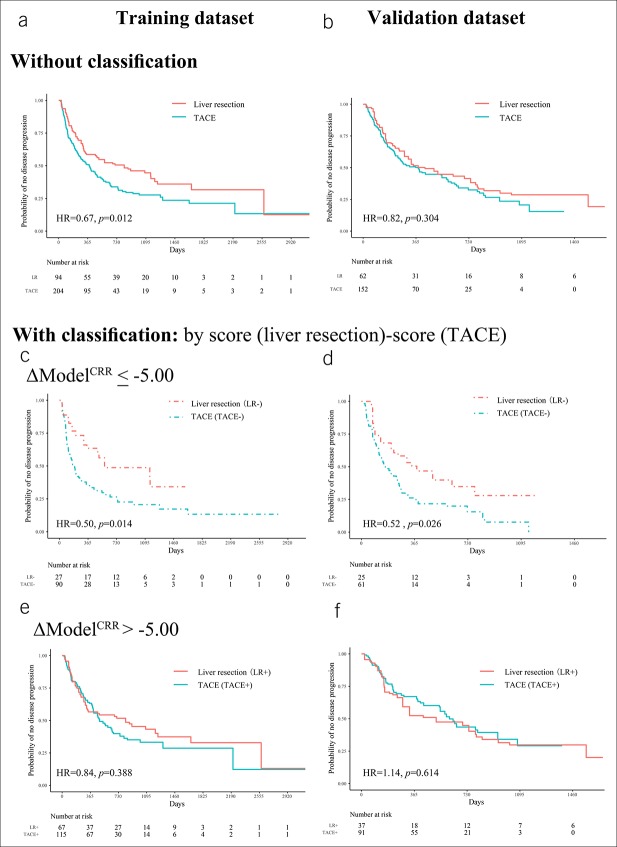
Kaplan-Meier analysis for PFS. Without classification, the PFS differences between LR and TACE are inconsistent in the training (**a**) and validation (**b**) data sets. After classification based on ΔModel^CRR^, for patients with ΔModel^CRR^ ≤ −5.00, LR demonstrated better results (**c** and **d**). For patients with ΔModel^CRR^ > −5.00, LR and TACE had similar PFS (**e** and **f**). PFS, progression-free survival; TACE transarterial chemoembolization; Model^CRR^ included clinical factors (C), radiological characteristics (R), and radiomic features (R). LR, liver resection; HR, hazard ratio; TACE, transarterial chemoembolization.

In the IPTW-weighted patients, the PFS difference between LR and TACE group was significant in the training data set: hazard ratio (HR) = 0.67 (95% confidence interval [CI]: 0.47–0.91), *P* = 0.012 (Figure 5a; see Table S4, Supplemental Digital Content 2, http://links.lww.com/CTG/A85); but not in the validation data set: HR = 0.82 (95% CI: 0.57–1.19), *P* = 0.304 (Figure 5b; see Table S4, Supplemental Digital Content 2, http://links.lww.com/CTG/A85). The inconsistency between data sets indicated that not all patients got PFS benefit from LR, and patients should be classified into subgroups for more reasonable treatment decision. Thus, we calculated the score difference of Model^CRR^ (ΔModel^CRR^) by the formula ΔModel^CRR^ = Model^CRR^ (LR score)−Model^CRR^ (TACE score). We used −5.00 as the threshold because we wanted to ensure PFS benefit for patients identified to be suitable for LR.

In patients with ΔModel^CRR^ ≤ −5.00 (LR− and TACE− subgroups), LR provided longer PFS. We obtained an HR = 0.50 (95% CI: 0.29–0.87), with *P* = 0.014 in the training data set (Figure 5c; see Table S4, Supplemental Digital Content 2, http://links.lww.com/CTG/A85), and HR = 0.52 (95% CI: 0.29–0.93), with *P* = 0.026 in the validation data set (Figure 5d; see Table S4, Supplemental Digital Content 2, http://links.lww.com/CTG/A85). Thus, LR might be the first option for these patients.

In patients with ΔModel^CRR^ > −5.00 (LR+ and TACE+ subgroups), there were no differences in PFS. In the training data set, the HR was 0.84 (95% CI: 0.57–1.25), with *P* = 0.388 (Figure 5e; see Table S4, Supplemental Digital Content 2, http://links.lww.com/CTG/A85); in the validation data set, the HR was 1.14 (95% CI: 0.69–1.85), with *P* = 0.614 (Figure 5f; see Table S4, Supplemental Digital Content 2, http://links.lww.com/CTG/A85). Thus, TACE might be a better choice because it was less invasive and did not cause decreased PFS.

**Figure 6.**
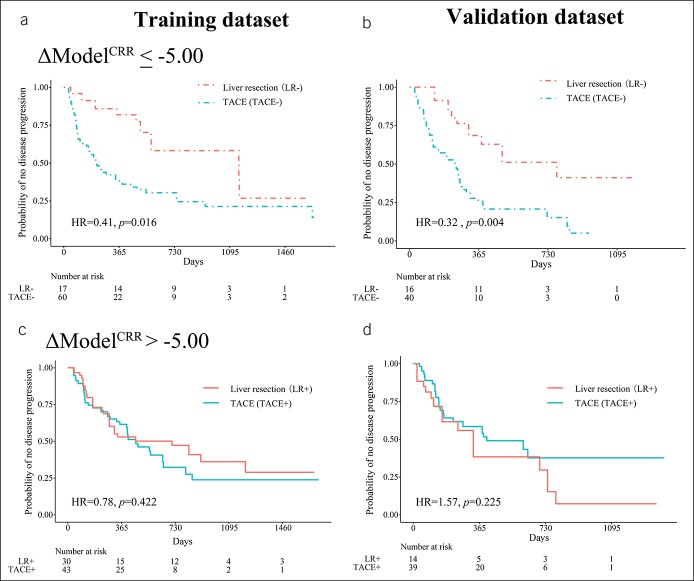
Kaplan-Meier analysis for PFS in BCLC stages AB and B. In the LR− and TACE− subgroups, the PFS difference had statistical differences in the training data set (**a**) and the validation data set (**b**). In the LR+ and TACE+ group, there were no statistical differences both in the training data set (**c**) and the validation data set (**d**). PFS, progression-free survival; BCLC, Barcelona Clinic Liver Cancer; LR, liver resection; TACE, transarterial chemoembolization.

**Figure 7.**
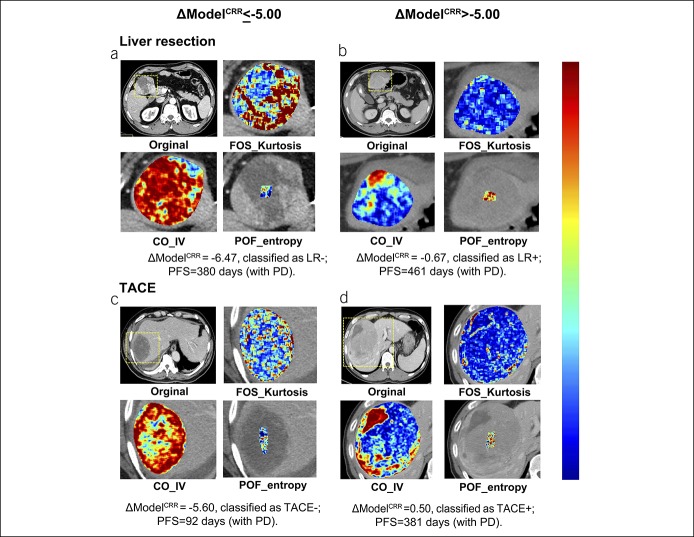
Representative patients. All of the 4 patients have single HCC >5 cm. (**a**) A 61-year-old male patient initially treated by LR, classified as LR−. (**b**) A 53-year-old male patient initially treated by LR, classified as LR+. (**c**) A 71-year-old man initially treated by TACE, classified as TACE−. (**d**) A 45-year-old man initially treated by TACE, classified as TACE+. HCC, hepatocellular carcinoma; LR, liver resection; TACE, transarterial chemoembolization.

We tested whether the classification was still informative in patients without extrahepatic metastasis as in a previous study (3), and the subgroup demonstrated similar results (see Figure S6, Supplemental Digital Content 3, http://links.lww.com/CTG/A86). Even if we excluded patients in BCLC stages A and C, our model could still classify patients either for LR or TACE (Figure 6). In patients with ΔModel^CRR^ ≤ −5.00, LR provided longer PFS. We obtained an HR = 0.41 (95% CI: 0.20–0.85), with *P* = 0.016 in the training data set, and HR = 0.32 (95% CI: 0.14–0.70), with *P* = 0.004 in the validation data set. For patients with ΔModel^CRR^ > −5.00, there were no differences in PFS: training data set, HR = 0.78 (95% CI: 0.43–1.42), with *P* = 0.422, validation dataset, HR = 1.57 (95% CI: 0.76–3.23), with *P* = 0.225. Representative patients of the 4 subgroups are shown in Figure 7.

## DISCUSSION

In this study, based on IPTW-weighted data, we used a noninvasive model to predict PFS of LR and TACE, which had good discrimination and calibration in both training and validation data sets. Then, subdivided by the threshold of the score difference, for some patients, LR provided better PFS than TACE, which suggested LR to be a potential better choice for increased PFS. However, for the other patients, because LR and TACE had similar PFS, TACE seemed a better option to control unnecessary trauma and risks. These conclusions were especially useful for patients in BCLC stages AB and B, in which there were more controversies between the choice of LR and TACE.

The choice between LR and TACE mainly depends on the difference in survival benefit; however, the patients' preference between controlling trauma/risks and survival difference should also be considered (35). Thus, besides classification, doctors and patients also need to know the predicted PFS associated with each treatment. For example, with a 6-month predicted PFS difference, a young father may tend to accept LR, whereas a 60-year-old patient may want TACE to control sufferings. However, when the PFS difference is 1-year, both may choose LR as the first option. Thus, after knowing the exact PFS difference predicted by the nomogram, the doctors may adjust the classification threshold of −5.00 according to clinical situation and patients' preference between risks/trauma and PFS benefit.

Compared with previous studies on HCC prognosis (36), our study not only performed PFS prediction on both LR and TACE cohorts but also established a procedure to compare the PFS between LR and TACE. Besides traditional clinical factors, such as BCLC stage including maximum diameter and lesion number, we also combined radiological characteristics and radiomic features. Compared with other clinical factors such as alpha-fetoprotein, these factors facilitated data mining from conventional CT images and provided more information of intratumor heterogeneity and were included in the final model. Compared with previous studies on treatments decision (3,14), instead of based on “post-treatment regret of doctors”, our model used more objective PFS end point, and we constructed our model on IPTW-weighted data set, which increased the comparability between LR and TACE groups, ensuring the causal effect between treatments and PFS difference. Because all our factors could be extracted through noninvasive methods, no additional biopsy would be required. Thus, treatment decision could be assisted with minimal disturbance of current HCC management.

In this study, besides conventional radiomic features (17), we also included “peer-off” features indicating HCC heterogeneity from the outside to the inside. Similar to CT attenuation, radiomic features reflect the granular changes in radiological images. As we cannot provide a definite causal relationship between CT attenuation and pathological results, we also cannot explain radiomic features pathologically. However, we may provide an explanation for the identified features based on their extraction methods. FOS_Kurtosis is Kurtosis of first-order statistics (0) and represents the first-order features on an original image. If the kurtosis is >3, the distribution of the intensity is sharper than a normal distribution (Figure 4d). CO_IV is inverse variance of co-occurrence (1,2) and represents the co-occurrence of the textural features of inverse variance on the x-direction high-pass and the y-direction low-pass filtered image. CO_IV emphasizes more edge information in the x-direction, and greater inverse variance indicates a more heterogeneous texture (Figure 4e). In our study, we found that when CO_IV decreases, the PFS between LR and TACE decreased at different rates, so independent scores of CO_IV were assigned for LR and TACE separately. Finally, POF_entropy is a peer-off feature of entropy (9) and represents the peel-off feature entropy in the innermost layer. It measures the texture randomness or irregularity of the layer (Figure 4f).

Our study has some limitations. First, because we perform IPTW in the training and validation separately, giving them a certain degree of independence, the potential bias of the analysis procedures can be controlled. Still, considering the complexity of the issue, an external validation data set is needed to further test our model. Second, for the differences of HCC between Asia and the Western countries, validation in a non-Chinese population is needed before our conclusions are applied in western cohorts. Third, as patients are divided into 8 subgroups for PFS analysis, the sample size of the study population may be relatively limited, and we were unable to perform more detailed analysis according to different BCLC stages. But ΔModel^CRR^ still successfully classifies patients based on PFS difference, which may also be helpful in controlling unnecessary surgical trauma and risks while ensuring better treatment for patients. Hopefully, these shortcomings can be addressed with further international research and cooperation.

In conclusion, our individualized model predicts the PFS difference between LR and TACE and assists the treatment decision: in selected patients, LR can be used to increase PFS benefit; in the others, TACE can be preferable to avoid unnecessary trauma and risks.

## CONFLICTS OF INTEREST

**Guarantors of the article:** Jie Tian, PhD and Ligong Lu, MD.

**Specific author contributions:** Sirui Fu, MD, Jingwei Wei, PhD, Jie Zhang, MD, and Di Dong, PhD, contributed equally to this work. S.F., J.W., J.Z., and D.D. conceived and designed the project with supervision from J.T. and L.L. J.S., Y.L., X.L., X.C., X.H., and J.Y. acquired the data. J.Z. and J.Y. segmented the data manually. S.Z., D.G., X.H., and Z.L. extracted radiomic features. C.D. performed the statistical analysis. All authors were involved in drafting and reviewing the manuscript and approved the final manuscript for submission.

**Financial support:** This work was supported by the National Key R&D Program of China (Nos. 2017YFA0205200, 2017YFC1308700, 2017YFC1308701, 2017YFC1309100, and 2016CZYD0001), the National Natural Science Foundation of China (Nos. 81571785, 81771957, 81227901, 61231004, 81771924, 81501616, 81671851, 81527805, and 81501549), the Natural Science Foundation of Guangdong Province, China (Nos. 2016A030311055 and 2016A030313770), the Science and Technology Service Network Initiative of the Chinese Academy of Sciences (KFJ-SW-STS-160), the Instrument Developing Project of the Chinese Academy of Sciences (YZ201502), the Beijing Municipal Science and Technology Commission (Z161100002616022), and the Youth Innovation Promotion Association CAS.

**Potential competing interests:** None.

**Study Highlights**

**WHAT IS KNOWN**

✓ LR and TACE are first-line treatments with overlapped adaptation in HCC.
✓ Choice between them should be assisted by noninvasive models to avoid unnecessary biopsies.

**WHAT IS NEW HERE**

✓ Our model predicted and compared PFS between LR and TACE.
✓ Subdivided by the score difference, LR provides longer PFS than TACE in some patients.
✓ In the other patients, the 2 treatments provided similar PFS.

**TRANSLATIONAL IMPACT**

✓ Our model predicted PFS difference between LR and TACE and assisted in choosing the optimal treatment.

## Supplementary Material

SUPPLEMENTARY MATERIAL
