# Supplementary material for: Selection Between Liver Resection Versus Transarterial Chemoembolization in Hepatocellular Carcinoma: A Multicenter Study
Source: Clin Transl Gastroenterol. 2019 Aug 1;10(8):e00070. doi: 10.14309/ctg.0000000000000070 (PMC6736221; doi:10.14309/ctg.0000000000000070)
Supplement: SUPPLEMENTARY MATERIAL [file ct9-10-e00070-s001.docx]

**Text S1. Selection of the target lesion.**

In general, the target lesion selected met the following criteria: 1) measurable (lesions that could be accurately measured in at least one dimension on a spiral CT scan and 2) for multiple lesions, the target lesion was selected on the basis of size (those with the longest diameter) and suitability for accurate repeated measurements.

**Text S2. Detailed definitions of the quantitative radiomics features.**

The extracted radiomics features were divided into two groups: original-image-based and filtered-image-based feature groups. For the original-image-based feature group, the peel-off features, Gabor wavelet features, and morphological features were extracted. For filtered-image-based features, we decomposed the original image into four filtered images using a two-dimensional wavelet transformation and extracted the first-order statistics features and textural features. The digital phantom was published online (http://www.radiomics.net.cn/post/108). We extracted 708 designed features as following:

1. Original-image-based feature group
2. Peel-off features

The growth pattern for the tumor was such that it enlarged from an inside seed to an outside periphery. We defined the peel-off layer as the layer containing the tumor voxels from the outside to the inside, shrinking in a 3-dimensional way. Peel-off features were evaluated to reflect the different phenotypic expression for each peel-off layer. The following 10 peel-off features were extracted:

X was the 3-dimensional peel-off layer with N voxels,

P was the first-order histogram with N_l_ discrete intensity levels,

V was the volume, and A was the surface area.

1. Surface Area

The surface area was calculated by triangulation (i.e., dividing the surface into connected triangles) and was defined as follows:

$$A=\sum_{i=1}^{N} \frac{1}{2}\left| \mathbf{a}_{\mathbf{i}}\mathbf{b}_{\mathbf{i}}\times\mathbf{a}_{\mathbf{i}}\mathbf{c}_{\mathbf{i}} \right|$$

where N was the total number of triangles covering the surface, and a, b, and c were edge vectors of the triangles.

1. Entropy

$$entropy=\sum_{i=1}^{N_{l}} p\left( i \right){log}_{2}p(i)$$

2. Kurtosis

$$kurtosis=\frac{\frac{1}{N}\sum_{i=1}^{N} \left( X\left( i \right)-\bar{X} \right)^{4}}{\left( \sqrt{\frac{1}{N}\sum_{i=1}^{N} \left( X\left( i \right)-\bar{X} \right)^{2}} \right)^{2}}$$

where $\bar{X}$ was the mean of X.

1. Mean

$$mean=\frac{1}{N}\sum_{i}^{N} X\left( i \right)$$

2. Surface-to-volume ratio

$$surface to volume ratio=\frac{A}{V}$$

The volume (V) was determined by counting the number of pixels in the tumor region and multiplying this value by the voxel size.

1. Root mean square (RMS)

$$RMS=\sqrt{\frac{\sum_{i}^{N} X\left( i \right)^{2}}{N}}$$

2. Skewness

$$skewness=\frac{\frac{1}{N}\sum_{i=1}^{N} \left( X\left( i \right)-\bar{X} \right)^{3}}{\left( \sqrt{\frac{1}{N}\sum_{i=1}^{N} \left( X\left( i \right)-\bar{X} \right)^{2}} \right)^{3}}$$

where $\bar{X}$ was the mean of X.

1. Standard deviation

$$standard deviation=\left( \frac{1}{N-1}\sum_{i=1}^{N} \left( X\left( i \right)-\bar{X} \right)^{2} \right)^{\frac{1}{2}}$$

where $\bar{X}$ was the mean of X.

1. Uniformity

$$uniformity=\sum_{i=1}^{N} P\left( i \right)^{2}$$

2. Energy

$$\mathrm{energy}\mathbf{=}\sum_{i}^{N} X\left( i \right)^{2}$$

3. Gabor wavelet features

We used Gabor filter to perform edge detection in eight directions and five scales. Gabor magnitude texture representation (GMTR) and Gabor phase-based texture representation (GPTR) were captured based on the convolution in multi-directional and multi-scale Gabor wavelet filtered images. The Gabor wavelet features included GMTRmean, GMTRvariance, and GPTRentropy. X denoted the three-dimensional Gabor-filtered image matrix with N pixels and P the first-order histogram with N_l_ discrete intensity levels. The detailed features were defined as follows:

1. GMTRmean

$$Mean=\frac{1}{N}\sum_{i}^{N} X\left( i \right)$$

2. GMTRvariance

$$Variance=\frac{1}{N-1}\sum_{i}^{N} \left( X\left( i \right)-Mean \right)^{2}$$

3. GPTRentropy

$$Entropy=\sum_{i=1}^{\mathrm{Nl}} P\left( i \right)\log_{2} P\left( i \right)$$

4. Morphological features (Features related to HCC morphological diversity)

Computer-based features enabled the discrimination of subtle differences in textural heterogeneity between targeted clinical groups. Empirical features based on the experience of doctors or physicians also contained critical information related to physiological or prognostic outcomes. In this study, we defined six morphological features that reflected the morphological diversity of the tumor lesion.

- - 1. Tumor border clearness

LCFeature. FuzzyofBounday reflected the clearness of the lesion border based on the intensity difference in the inside and outside areas of the tumor circle. The fuzzier would be border when the intensity difference was smaller.

1. Necrosis area

LCFeature. NecrosisareaRatio represented the ratio between the necrosis and the lesion areas. The necrosis could be observed in CT images with intensities less than 30 HU. Thus, as the necrosis pixel, we included pixels with intensities less than 30 HU. We extracted this feature on the image only after the wavelet transform with low-pass filtering in both the x- and y-directions.

1. Shape of the tumor (circle shape)

LCFeature.ShapeofCircle reflected the growth pattern of the tumor; if its shape was similar to a circle, it might show better prognosis. This calculation was based on the three-dimensional curvatures of the voxels on the boundary. This defined the standard deviance as the final parameter for the regularity of the border.

1. The number of nodule heaves on the border

LCFeature. NumofHeave reflected bulgy nodules on the tumor border based on the detection of the odds ratio of regularly and irregularly growing areas. It simulated a circle to represent the lesion with the same area, and then it calculated the ratio of the area outside the circle and the lesion area.

1. Difference between the center and cavity

LCFeature. CenterToCavity tended to reflect the faster growing direction of the tumor. This feature was calculated based on the distance between the coordinates of the center and the cavity.

1. Mosaic area

LCFeature. Mosaic measured pixel intensities that were different from those of the tumor border. The intensity range for the tumor border was [125, 167], while the range for the mosaic area was [130, 167]. This searched the 20 pixels inside and outside the tumor border for the mosaic area.

1. Filtered-image-based Feature group

The original image set was decomposed into four filtered image sets using two-dimensional wavelet transformation with low- and high-pass functions on each image slice. First-order statistics and textural features were extracted.

1. First order statistics feature group

The first-order statistics features represented the distribution of voxel intensity within the filtered image. A set of 14 first-order statistics features were calculated. The X denoted the image matrix with N voxels, and P, the first-order histogram with N_l_ discrete intensity levels. The definition of the 14 features were as follows:

1. Energy

$$\mathrm{energy}\mathbf{=}\sum_{i}^{N} X\left( i \right)^{2}$$

2. Entropy

$$entropy=\sum_{i=1}^{N_{l}} P\left( i \right)\log_{2} P\left( i \right)$$

3. Kurtosis

$$kurtosis=\frac{\frac{1}{N}\sum_{i=1}^{N} \left( X\left( i \right)-\bar{X} \right)^{4}}{\left( \sqrt{\frac{1}{N}\sum_{i=1}^{N} \left( X\left( i \right)-\bar{X} \right)^{2}} \right)^{2}}$$

where $\bar{X}$ was the mean of X.

1. Maximum

The maximum intensity value of X.

1. Mean

$$mean=\frac{1}{N}\sum_{i}^{N} X\left( i \right)$$

2. Mean absolute deviation

The mean of the absolute deviations of all the voxel intensities around the mean intensity value.

1. Median

The median intensity value of X.

1. Minimum

The minimum intensity value of X.

1. Range

The range of intensity values of X.

1. Root mean square (RMS)

$$RMS=\sqrt{\frac{\sum_{i}^{N} X\left( i \right)^{2}}{N}}$$

2. Skewness

$$skewness=\frac{\frac{1}{N}\sum_{i=1}^{N} \left( X\left( i \right)-\bar{X} \right)^{3}}{\left( \sqrt{\frac{1}{N}\sum_{i=1}^{N} \left( X\left( i \right)-\bar{X} \right)^{2}} \right)^{3}}$$

3. Standard deviation

$$standard deviation=\left( \frac{1}{N-1}\sum_{i=1}^{N} \left( X\left( i \right)-\bar{X} \right)^{2} \right)^{\frac{1}{2}}$$

4. Uniformity

$$uniformity=\sum_{i=1}^{N_{t}} P\left( i \right)^{2}$$

5. Variance

$$variance=\frac{1}{N-1}\sum_{i=1}^{N} \left( X\left( i \right)-\bar{X} \right)^{2}$$

6. Textural feature group

Textural features described the intrinsic heterogeneous texture of the tumor lesion. The Gray Level Co-occurrence Matrix (GLCM) and the Gray Level Run-Length Matrix (GLRLM) were constructed after discretizing the regions of interest by resampling the voxel intensities into equally spaced bins using a bin-width of 25 Hounsfield Units (HU).

1. GLCM Feature Group

GLCM represented second-order statistical characteristics of the image. We used P(i, j; θ, α) to denote the GLCM where the (i, j)^th^ element represented two pixels with intensity levels i and j occurring at a distance of θ=1 in the direction α (0°, 45°, 90°, 135°).

N_g_ was the number of discrete intensity levels in the image,

μ was the mean of P(i, j),

$p_{x}\left( i \right)=\sum_{j=1}^{N_{g}} P(i,j)$ depicted the marginal row probabilities,

$p_{y}\left( i \right)=\sum_{i=1}^{N_{g}} P(i,j)$ depicted the marginal column probabilities,

$\mu_{x}$(i) was the mean of $P_{x}$(i),

$\mu_{y}$(i) was the mean of $P_{y}$(i),

$\delta_{x}$(i) was the standard deviation of $P_{x}$(i),

$\delta_{y}$(i) was the standard deviation of $P_{y}$(i),

$p_{x+y}\left( k \right)=\sum_{i=1}^{N_{g}} \sum_{j=1}^{N_{g}} P\left( i,j \right)$, i+j=k,k=2,3,…,2 N_g_,

$p_{x-y}\left( k \right)=\sum_{i=1}^{N_{g}} \sum_{j=1}^{N_{g}} P\left( i,j \right)$, |i-j|=k,k=2,3,…,2 N_g_,

$HX=-\sum_{i=1}^{N_{g}} p_{x}\left( i \right)\log_{2} \left[ p_{x}\left( i \right) \right]$ was the entropy of p_x_,

$HY=-\sum_{i=1}^{N_{g}} p_{y}(i)\log_{2} \left[ p_{y}(i) \right]$ was the entropy of p_y_,

$H=-\sum_{i=1}^{N_{g}} \sum_{j=1}^{N_{g}} P(i,j)\log_{2} \left[ P(i,j) \right]$ was the entropy of P(i,j),

$HXY1=-\sum_{i=1}^{N_{g}} \sum_{j=1}^{N_{g}} P(i,j)\log(p_{x}(i)p_{y}(j))$,

HXY2= $-\sum_{i=1}^{N_{g}} \sum_{j=1}^{N_{g}} p_{x}\left( i \right)p_{y}(i)\log(p_{x}(i)p_{y}(j))$,

1. Autocorrelation

$$autocorrelation=\sum_{i=1}^{N_{g}} \sum_{j=1}^{N_{g}} ijP(i,j)$$

2. Cluster Prominence

$$cluster prominence=\sum_{i=1}^{N_{g}} \sum_{j=1}^{N_{g}} \left( i+j-\mu_{x}\left( i \right)-\mu_{y}\left( j \right) \right)^{4}P(i,j)$$

3. Cluster Shade

$$cluster shade=\sum_{i=1}^{N_{g}} \sum_{j=1}^{N_{g}} \left( i+j-\mu_{x}\left( i \right)-\mu_{y}\left( j \right) \right)^{3}P(i,j)$$

4. Cluster Tendency

$$cluster tendency=\sum_{i=1}^{N_{g}} \sum_{j=1}^{N_{g}} \left( i+j-\mu_{x}\left( i \right)-\mu_{y}\left( j \right) \right)^{2}P(i,j)$$

5. Contrast

$$contrast=\sum_{i=1}^{N_{g}} \sum_{j=1}^{N_{g}} \left| i-j \right|^{2}P(i,j)$$

6. Correlation

$$correlation=\frac{\sum_{i=1}^{N_{g}} \sum_{j=1}^{N_{g}} \mathrm{ijP}\left( i,j \right)-\mu_{i}\left( i \right)\mu_{j}\left( j \right)}{\sigma_{x}(i)\sigma_{y}(j)}$$

7. Difference entropy

$$difference entropy=\sum_{i=0}^{N_{g}-1} P_{x-y}(i)\log_{2} \left[ P_{x-y}(i) \right]$$

8. Dissimilarity

$$dissimilarity=\sum_{i=1}^{N_{g}} \sum_{j=1}^{N_{g}} \left| i-j \right|P(i,j)$$

9. Energy

$$energy=\sum_{i=1}^{N_{g}} \sum_{j=1}^{N_{g}} {P(i,j)}^{2}$$

10. Entropy

$$entropy=-\sum_{i=1}^{N_{g}} \sum_{j=1}^{N_{g}} P(i,j)\log_{2} \left[ P(i,j) \right]$$

11. Homogeneity 1

$$homogeneity 1=\sum_{i=1}^{N_{g}} \sum_{j=1}^{N_{g}} \frac{P(i,j)}{1+\left| i-j \right|}$$

12. Homogeneity 2

$$homogeneity 2=\sum_{i=1}^{N_{g}} \sum_{j=1}^{N_{g}} \frac{P(i,j)}{1+\left| i-j \right|^{2}}$$

13. Informational measure of correlation 1 (IMC 1)

$$IMC1=\frac{HXY-HXY1}{\max\left\{ HX,HY \right\}}$$

14. Information measure of correlation 2 (IMC 2)

$$IMC2=\sqrt{1-e^{-2\left( HXY2-HXY \right)}}$$

15. Inverse Difference Moment Normalized (IDMN)

$$IDMN=\sum_{i=1}^{N_{g}} \sum_{j=1}^{N_{g}} \frac{P(i,j)}{1+(\frac{\left| i-j \right|^{2}}{N^{2}})}$$

16. Inverse Difference Normalized (IDN)

$$IDN=\sum_{i=1}^{N_{g}} \sum_{j=1}^{N_{g}} \frac{P(i,j)}{1+(\frac{\left| i-j \right|}{N})}$$

17. Inverse variance

$$inverse variance=\sum_{i=1}^{N_{g}} \sum_{j=1}^{N_{g}} \frac{P(i,j)}{\left| i-j \right|^{2}}$$

18. Maximum Probability

$$maximum probability=max\left\{ P(i,j) \right\}$$

19. Sum average

$$sum average=\sum_{i=2}^{{2N}_{g}} \left[ iP_{x+y}(i) \right]$$

20. Sum entropy

$$sum entropy=-\sum_{i=2}^{{2N}_{g}} P_{x+y}(i)\log_{2} \left[ P_{x+y}(i) \right]$$

21. Sum variance

$$sum variance=\sum_{i=2}^{{2N}_{g}} {(i-SE)}^{2}P_{x+y}(i)$$

22. Variance

$$variance=\sum_{i=1}^{N_{g}} \sum_{j=1}^{N_{g}} \left( i-\mu\right)^{2}P(i,j)$$

23. GLRLM feature group

GLRLM quantified the gray level runs of the image. We used P(i, j| θ) to denote the GLRLM where the (i, j)^th^ element represented a pixel with a gray level i occurring consecutively at j times in the direction θ (0°, 45°, 90°, 135°).

N_g_ was the number of discrete intensity levels in the image，

N_r_ was the number of different run lengths,

N_p_ was the number of voxels in the image.

1. Short Run Emphasis (SRE)

$$SRE=\frac{\sum_{i=1}^{N_{g}} \sum_{j=1}^{N_{r}} \left[ \frac{\rho(i,j|\theta)}{j^{2}} \right]}{\sum_{i=1}^{N_{g}} \sum_{j=1}^{N_{r}} \rho(i,j|\theta)}$$

2. Long Run Emphasis (LRE)

$$LRE=\frac{\sum_{i=1}^{N_{g}} \sum_{j=1}^{N_{r}} j^{2}\rho(i,j|\theta)}{\sum_{i=1}^{N_{g}} \sum_{j=1}^{N_{r}} \rho(i,j|\theta)}$$

3. Gray Level Non-Uniformity (GLN)

$$GLN=\frac{\sum_{i=1}^{N_{g}} \left[ \sum_{j=1}^{N_{r}} \rho(i,j|\theta) \right]^{2}}{\sum_{i=1}^{N_{g}} \sum_{j=1}^{N_{r}} \rho(i,j|\theta)}$$

4. Run Length Non-Uniformity (RLN)

$$RLN=\frac{\sum_{j=1}^{N_{r}} \left[ \sum_{i=1}^{N_{g}} \rho(i,j|\theta) \right]^{2}}{\sum_{i=1}^{N_{g}} \sum_{j=1}^{N_{r}} \rho(i,j|\theta)}$$

5. Run Percentage (RP)

$$RP=\sum_{i=1}^{N_{g}} \sum_{j=1}^{N_{r}} \frac{\rho(i,j|\theta)}{N_{\rho}}$$

6. Low Gray Level Run Emphasis (LGLRE)

$$LGLRE=\frac{\sum_{i=1}^{N_{g}} \sum_{j=1}^{N_{r}} \left[ \frac{\rho(i,j|\theta)}{i^{2}} \right]}{\sum_{i=1}^{N_{g}} \sum_{j=1}^{N_{r}} \rho(i,j|\theta)}$$

7. High Gray Level Run Emphasis (HGLRE)

$$HGLRE=\frac{\sum_{i=1}^{N_{g}} \sum_{j=1}^{N_{r}} i^{2}\rho(i,j|\theta)}{\sum_{i=1}^{N_{g}} \sum_{j=1}^{N_{r}} \rho(i,j|\theta)}$$

8. Short Run Low Gray Level Emphasis (SRLGLE)

$$SRLGLE=\frac{\sum_{i=1}^{N_{g}} \sum_{j=1}^{N_{r}} \left[ \frac{\rho(i,j|\theta)}{i^{2}j^{2}} \right]}{\sum_{i=1}^{N_{g}} \sum_{j=1}^{N_{r}} \rho(i,j|\theta)}$$

9. Short Run High Gray Level Emphasis (SRHGLE)

$$SRHGLE=\frac{\sum_{i=1}^{N_{g}} \sum_{j=1}^{N_{r}} \left[ \frac{\rho(i,j|\theta)i^{2}}{j^{2}} \right]}{\sum_{i=1}^{N_{g}} \sum_{j=1}^{N_{r}} \rho(i,j|\theta)}$$

10. Long Run Low Gray Level Emphasis (LRLGLE)

$$LRLGLE=\frac{\sum_{i=1}^{N_{g}} \sum_{j=1}^{N_{r}} \left[ \frac{\rho(i,j|\theta)j^{2}}{i^{2}} \right]}{\sum_{i=1}^{N_{g}} \sum_{j=1}^{N_{r}} \rho(i,j|\theta)}$$

11. Long Run High Gray Level Emphasis (LRHGLE)

$$LRHGLE=\frac{\sum_{i=1}^{N_{g}} \sum_{j=1}^{N_{r}} \rho(i,j|\theta)i^{2}j^{2}}{\sum_{i=1}^{N_{g}} \sum_{j=1}^{N_{r}} \rho(i,j|\theta)}$$

12. Energy

Energy =$\sum_{i}^{N_{g}} p(i,j|\theta)^{2}$

1. Intensity mean

Intensity mean = $\frac{1}{N_{g}}\sum_{i}^{N_{g}} p(i,j|\theta)$

1. Run-length mean

Run-length mean = $\frac{1}{N_{r}}\sum_{i}^{N_{r}} p(i,j|\theta)$

**Text S3. Morphologic perturbations of radiomic features.**

For morphologic perturbations of radiomic features, we perturbed the original region of interest by 1) shifting at 3-pixel increments in left, right, up, and down directions; 2) morphologically opening or closing a 3🞨 3 square window; and 3) rotating the image by 3° in a clockwise or counterclockwise direction. We then assessed the intra-class correlation (ICC) between one original and eight perturbed ROIs.

**Text S4. Detailed method of inverse probability of treatment weighting (IPTW).**

Age, alanine aminotransferase, sex, hepatitis B virus, hepatitis C virus, cirrhosis, Child-Pugh class, Barcelona Clinic Liver Cancer (BCLC), extrahepatic metastasis, macrovascular invasion, and alpha-fetoprotein levels were considered in propensity score model development. As in BCLC stage C, extrahepatic metastasis and macrovascular invasion might each have a different influence on treatment selection; for instance, macrovascular could severely deteriorate liver function and increase the risk of liver resection. Therefore, extrahepatic metastasis and macrovascular invasion were also used as independent factors in IPTW. For both training and validation datasets, the propensity score model was developed using the following sequential steps. First, the factors were all included in the logistic regression model to predict the usage of liver resection. Second, for each continuous variable, the model in step one was compared with a model that incorporated the restricted cubic spline function with three knots for all continuous variables. Non-significant cubic splines were excluded. Third, we examined the potential interactions between predictor variables by the Least Absolute Shrinkage and Selection Operator method. Due to a large number of potential interactions, only two-way interactions were considered. Interactions found to be significant were then retained for subsequent steps. Fourth, we developed a full model including all main effects, cubic-spline representations for continuous variables and interactions that were identified in the second and third steps. This final model was used to estimate the propensity scores.

Subjects were weighted by using IPTW based on the propensity scores to create a synthetic sample in which treatment assignment was independent of considered baseline covariates. Stabilized weights (1) were used instead of the conventional weights. Balance between treatment groups in the weighted sample was assessed following the methods introduced as reported (2). Prediction models were developed based on the weighted dataset.

**Text S5. Comparability between liver resection and TACE groups increased after IPTW.**

IPTW was used to balance baseline differences as reported (2,3). Considering the potential problem of overfitting (4), we randomly separated the database at a 3:2 ratio to create training and validation datasets as reported by previous studies (5,6). Before model construction, we performed IPTW to ensure comparability between the liver resection and TACE groups. Before IPTW, for the training dataset, the mean propensity score in the liver resection group was 0.28 ± 0.15 (median=0.25, Q25−Q75: 0.16−0.42), and the mean propensity score in the TACE group was 0.40 ± 0.15 (median=0.43, Q25−Q75: 0.29−0.51). For the validation dataset, the mean propensity score in the liver resection group was 0.25 ± 0.16 (median=0.24, Q25−Q75: 0.12−0.36), and the mean propensity score in the TACE group was 0.41 ± 0.18 (median=0.39, Q25−Q75: 0.28−0.52). After weighting, good balance (with most standardized differences <10%) was achieved in both training and validation datasets. For hepatitis C virus, although the standardized differences between the groups were greater than 10% after weighting, the absolute differences were small (0.9% and 0.0% for training and validation datasets, respectively).

**Text S6. Testing the necessity of applying modified BCLC and stage AB.**

We compared the AUC and calibration between Model^CRR^ (applying modified BCLC and stage AB) and Model^original^ (applying the original BCLC stage). The results showed that although the AUCs had no statistical differences (Fig. S4), Model^CRR^ had better calibration than Model^original^ (Fig. S5).

**References:**

1. Cole SR, Hernan MA. Constructing inverse probability weights for marginal structural models. Am J Epidemiol 2008;168:656-64.

2. Austin PC, Stuart EA. Moving towards best practice when using inverse probability of treatment weighting (IPTW) using the propensity score to estimate causal treatment effects in observational studies. Stat Med 2015;34:3661-79.

3. Wahl DR, Stenmark MH, Tao Y, et al. Outcomes After Stereotactic Body Radiotherapy or Radiofrequency Ablation for Hepatocellular Carcinoma. J Clin Oncol 2016;34:452-9.

4. Cook JA, Ranstam J. Overfitting. Br J Surg 2016;103:1814.

5. Farinati F, Vitale A, Spolverato G, et al. Development and Validation of a New Prognostic System for Patients with Hepatocellular Carcinoma. Plos Med 2016;13:e1002006.

6. Xu L, Peng ZW, Chen MS, et al. Prognostic nomogram for patients with unresectable hepatocellular carcinoma after transcatheter arterial chemoembolization. J Hepatol 2015;63:122-30.
