# Supplementary material for: Selection Between Liver Resection Versus Transarterial Chemoembolization in Hepatocellular Carcinoma: A Multicenter Study
Source: Clin Transl Gastroenterol. 2019 Aug 1;10(8):e00070. doi: 10.14309/ctg.0000000000000070 (PMC6736221; doi:10.14309/ctg.0000000000000070)
Supplement: SUPPLEMENTARY MATERIAL [file ct9-10-e00070-s002.docx]

**Table S1. The major parameters of CT image acquisition in the participating hospitals.**

| **Hospitals** | **Scanner** | **TV (kV)** | **TC (mA)** | **RT (s)** | **DC (mm)** | **FOV (mm)** | **PM** | **Reconstruction** | **ST (mm)** |
| --- | --- | --- | --- | --- | --- | --- | --- | --- | --- |
| **NFH** | Philips Brilliance | 120 | 142 | 0.75 | 128×0.625 | 300×300 | 512×512 | Filter sharp (C) | 5 mm |
| **SPH** | Philips Brilliance | 120 | 250 | 0.50 | 64×0.625 | 500×500 | 1024×1024 | Filter sharp (C) | 5 mm |
| **YPH** | Philips Brilliance | 120 | 300 | 0.75 | 64×0.625 | 350×350 | 512×512 | Filter sharp (C) | 2 mm |
| **ZCPH** | Philips Brilliance | 120 | 250 | 0.50 | 128×0.625 | 350×350 | 512×512 | Filter Standard(B) | 5 mm |
| **ZPH** | Siemens Somatom Definition Flash | 120 | 160 | 0.50 | 64×0.625 | 350×350 | 512×512 | Filter sharp (C) | 2 mm  5 mm |
| TV: tube voltage; TC: tube current; RT: rotation time; DC: detector collimation; FOV: field of view; PM: pixel matrix; ST: slice thickness; NFH: Nanfang Hospital; SPH: Shenzhen People’s Hospital; YPH: Yangjiang People’s Hospital; ZCPH: Zhongshan City People’s Hospital; ZPH: Zhuhai People’s Hospital | | | | | | | | | |

**Table S2 Standardized differences between liver resection and TACE groups after weighting by IPTW.**

|  | **Training dataset (%)** | | **Validation dataset (%)** | |
| --- | --- | --- | --- | --- |
|  | Before IPTW | After IPTW | Before IPTW | After IPTW |
| **Age** | 20.52 | 4.05 | 34.97 | 7.37 |
| **ALT** | 6.82 | -4.34 | 33.04 | 5.76 |
| **Age^2^** | 21.55 | 4.36 | 34.99 | 8.72 |
| **ALT^2^** | 3.73 | -4.91 | 31.1 | 8.61 |
| **Age*ALT** | 11.25 | -3.00 | 41.54 | 10.81 |
| **Sex (Female)** | 22.77 | 6.71 | -0.98 | -1.70 |
| **HBV** | -5.10 | 4.04 | -1.09 | -1.98 |
| **HCV** | 17.23 | 14.19 | 16.28 | 13.64 |
| **Cirrhosis** | 10.85 | 0.47 | 11.9 | -0.93 |
| **Child-Pugh class** |  |  |  |  |
| A | -7.61 | 3.00 | -41.61 | -8.91 |
| B | 7.61 | -3.00 | 41.61 | 8.91 |
| **BCLC stage** |  |  |  |  |
| 0 | -13.07 | -1.89 | 0.56 | -7.03 |
| A | -37.32 | -0.39 | -43.03 | -1.98 |
| AB | -24.52 | -2.21 | -2.01 | -0.91 |
| B | 44.59 | 4.35 | 11.80 | 8.62 |
| C | 36.7 | -0.04 | 39.38 | -0.12 |
| **Macrovascular invasion** | 35.72 | 1.84 | 30.27 | -1.17 |
| **Extrahepatic metastasis** | 17.34 | 2.71 | 30.15 | 7.95 |
| **AFP level (ng/ml, n)** |  |  |  |  |
| <25 | -6.28 | -5.76 | 5.05 | 7.15 |
| 25–400 | -0.70 | 5.42 | -22.96 | -10.29 |
| >400 | 6.78 | 0.99 | 17.14 | 2.24 |
| ^2^square; *factors with an interaction | | | | |

**Table S3. Pairwise comparison by likelihood ratio among Model^CR^, Model^R^, Model^CRR^, ITA.LI.CA, and CLIP (*p* value).**

|  | **Model^CR^** | **Model^R^** | **Model^CRR^** | **ITA.LI.CA** | **CLIP** |
| --- | --- | --- | --- | --- | --- |
| **Training dataset** | | | | | |
| **Model^CR^** | - | <0.001 | 0.009 | <0.001 | <0.001 |
| **Model^R^** | <0.001 | - | <0.001 | 1.000 | 0.532 |
| **Model^CRR^** | 0.009 | <0.001 | - | <0.001 | <0.001 |
| **ITA.LI.CA** | <0.001 | 1.000 | <0.001 | - | <0.001 |
| **CLIP** | <0.001 | 0.532 | <0.001 | <0.001 | - |
| **Validation dataset** | | | | | |
| **Model^CR^** | - | 0.279 | 0.001 | <0.001 | <0.001 |
| **Model^R^** | 0.279 | - | <0.001 | <0.001 | <0.001 |
| **Model^CRR^** | 0.001 | <0.001 | - | <0.001 | <0.001 |
| **ITA.LI.CA** | <0.001 | <0.001 | <0.001 | <0.001 | <0.001 |
| **CLIP** | <0.001 | <0.001 | <0.001 | <0.001 | <0.001 |
| Model^CR^: model consisting of clinical factors and radiological characteristics; Model^R^: model consisting of radiomic features; Model^CRR^: model consisting of clinical factors, radiological characteristics, and radiomic features; | | | | | |

**Table S4. Kaplan-Meier analysis for PFS in IPTW-weighted datasets.**

|  | **Training dataset** | | | | | **Validation dataset** | | | | |
| --- | --- | --- | --- | --- | --- | --- | --- | --- | --- | --- |
|  | **Cases** | **PD** | **mPFS (Days)** | **HR (95%CI)** | ***p*** | **Cases** | **PD** | **mPFS (Days)** | **HR (95%CI)** | ***p*** |
| **Without classification** |  |  |  | 0.67 (0.47–0.91) | 0.012 |  |  |  | 0.82 (0.57–1.19) | 0.304 |
| LR | 94.3 | 54.3 | 823 |  |  | 62.0 | 40.9 | 427 |  |  |
| TACE | 204.4 | 137.7 | 385 |  |  | 152.0 | 101.2 | 381 |  |  |
| **With classification** |  |  |  |  |  |  |  |  |  |  |
| ΔModel^CRR^<-5.00 |  |  |  | 0.50 (0.29–0.87) | 0.014 |  |  |  | 0.52 (0.29–0.93) | 0.026 |
| LR- | 27.3 | 15.8 | 576 |  |  | 24.5 | 15.1 | 380 |  |  |
| TACE- | 89.5 | 67.8 | 185 |  |  | 61.3 | 52.1 | 164 |  |  |
| ΔModel^CRR^>-5.00 |  |  |  | 0.84 (0.57–1.25) | 0.388 |  |  |  | 1.14 (0.69–1.85) | 0.614 |
| LR+: | 67.0 | 38.5 | 823 |  |  | 37.4 | 25.8 | 516 |  |  |
| TACE+ | 114.8 | 69.9 | 485 |  |  | 91.0 | 49.2 | 608 |  |  |
| mPFS: median progression-free survival; LR: liver resection | | | | |  |  |  |  |  |  |
