# Supplementary material for: Selection Between Liver Resection Versus Transarterial Chemoembolization in Hepatocellular Carcinoma: A Multicenter Study
Source: Clin Transl Gastroenterol. 2019 Aug 1;10(8):e00070. doi: 10.14309/ctg.0000000000000070 (PMC6736221; doi:10.14309/ctg.0000000000000070)
Supplement: SUPPLEMENTARY MATERIAL [file ct9-10-e00070-s003.pptx]

## Slide 1
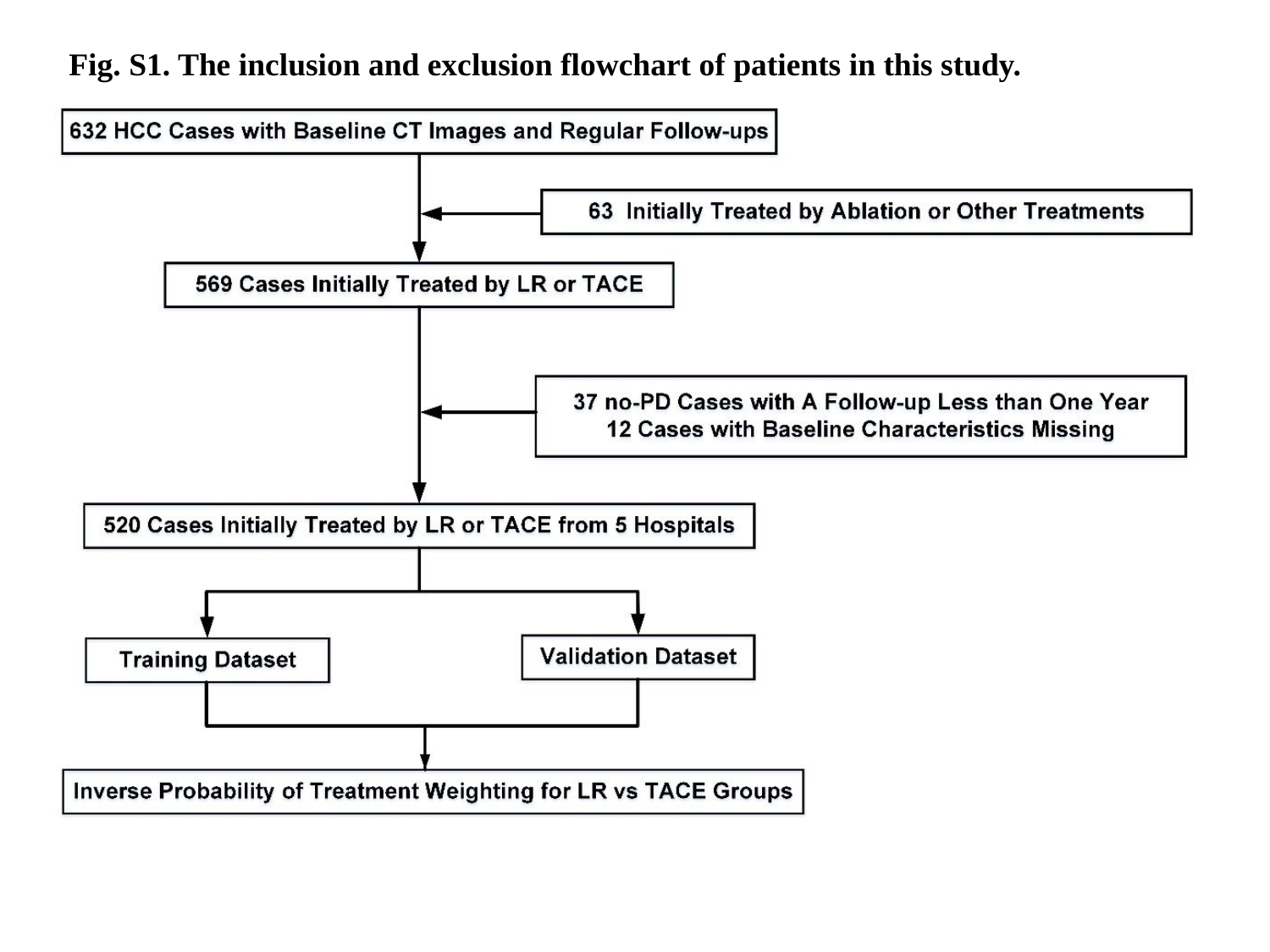

Fig. S1. The inclusion and exclusion flowchart of patients in this study.

## Slide 2
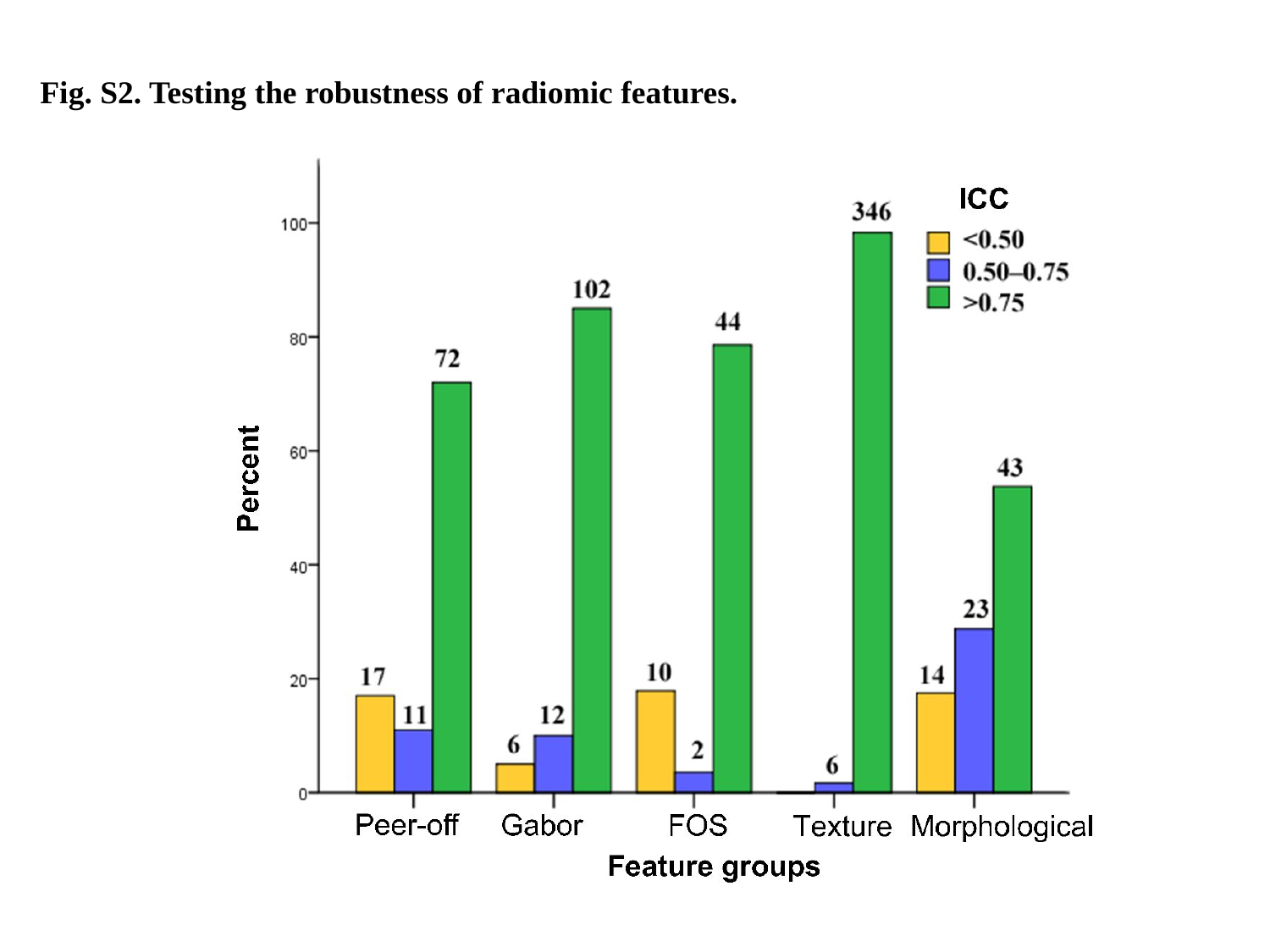

Fig. S2. Testing the robustness of radiomic features.

## Slide 3
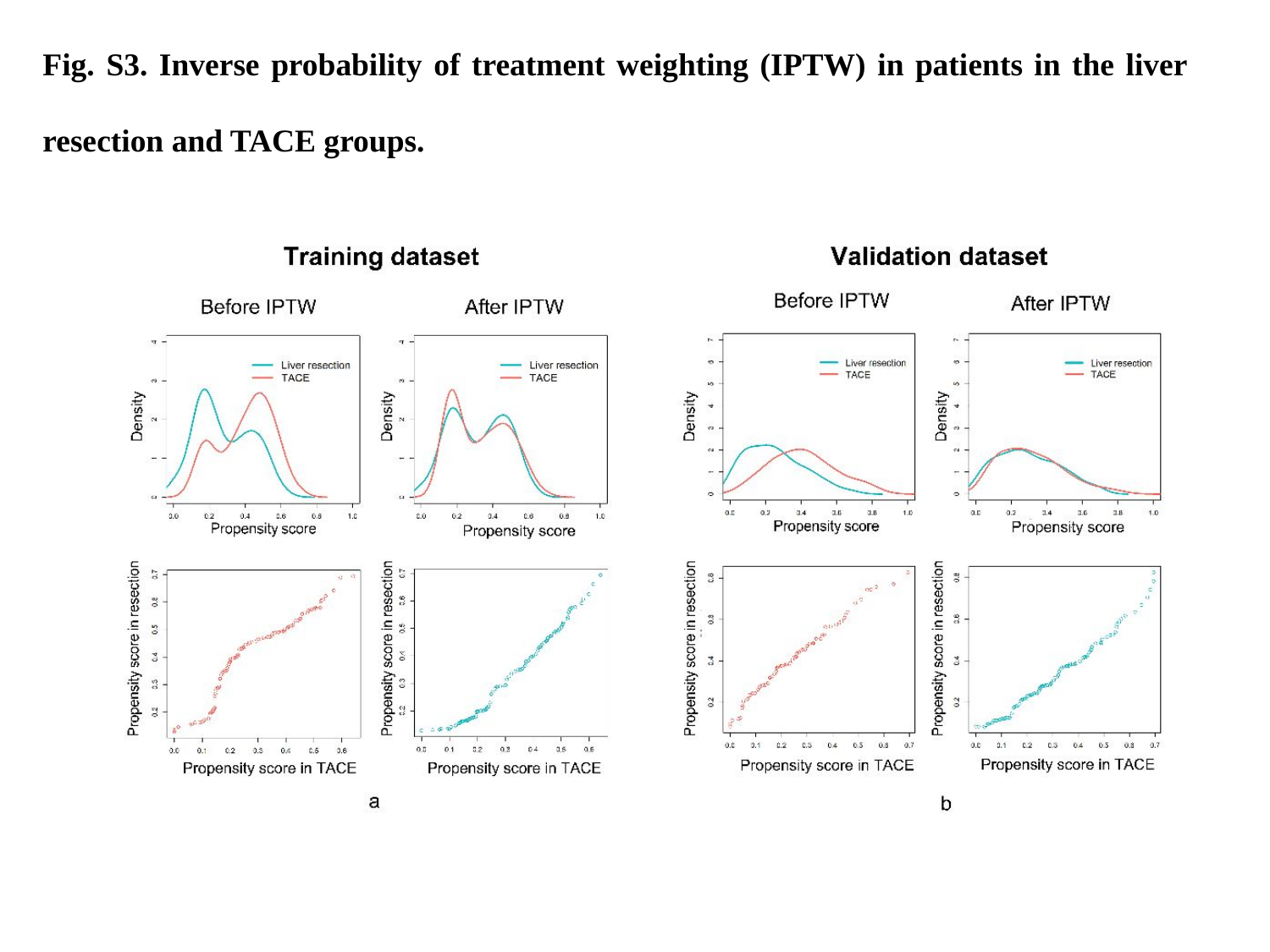

Fig. S3. Inverse probability of treatment weighting (IPTW) in patients in the liver resection and TACE groups.

## Slide 4
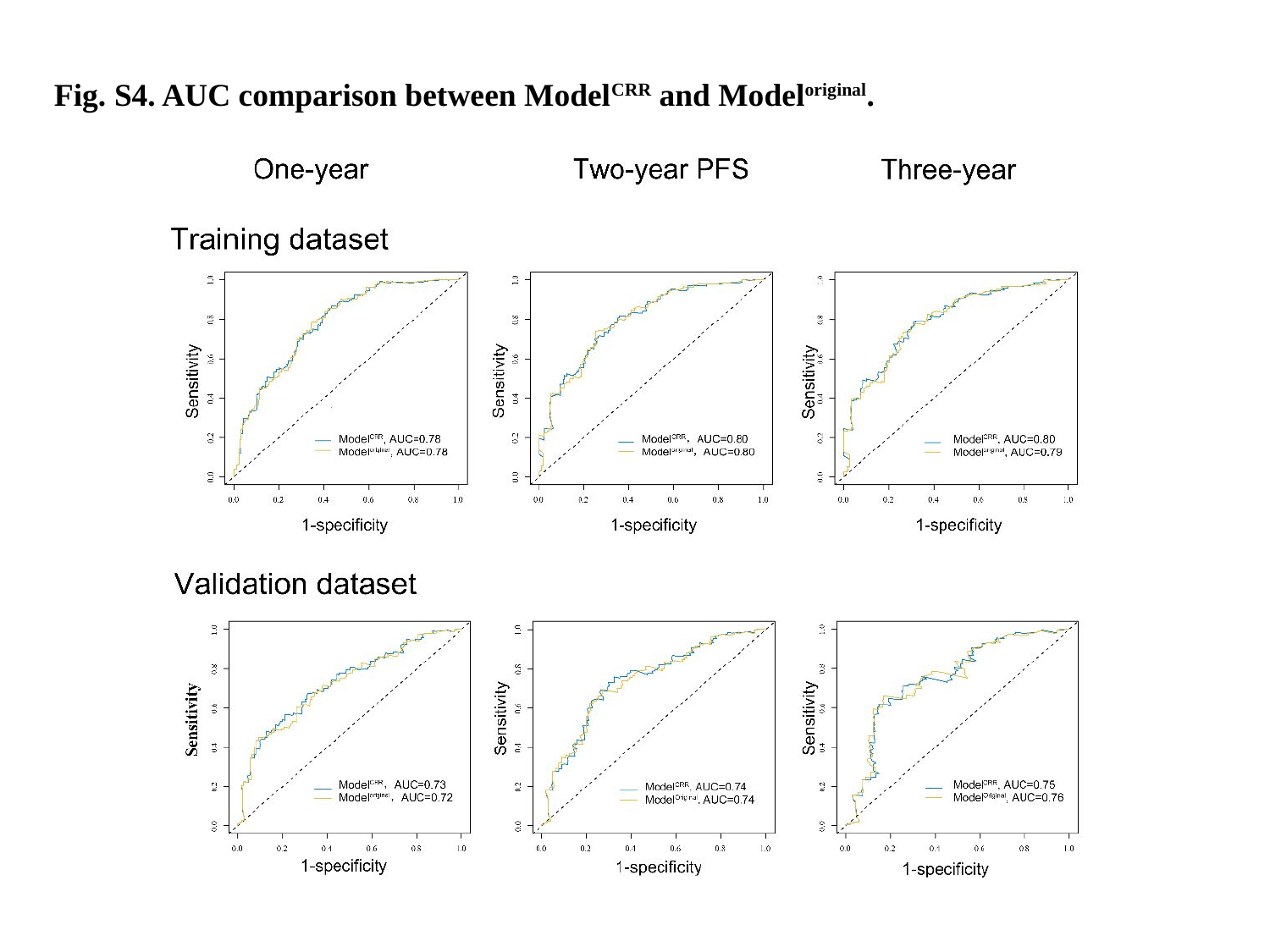

Fig. S4. AUC comparison between ModelCRR and Modeloriginal.

## Slide 5
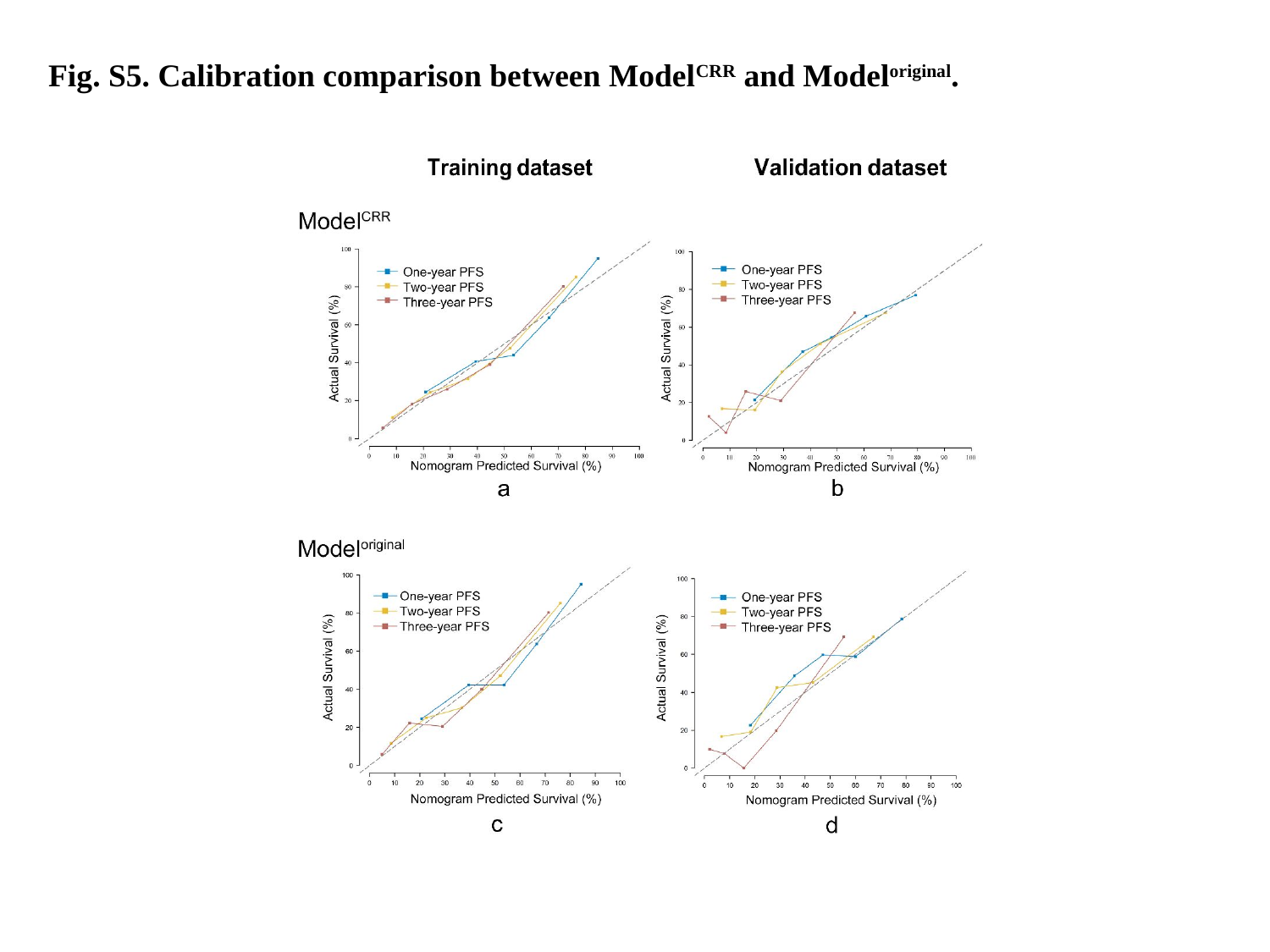

Fig. S5. Calibration comparison between ModelCRR and Modeloriginal.

## Slide 6
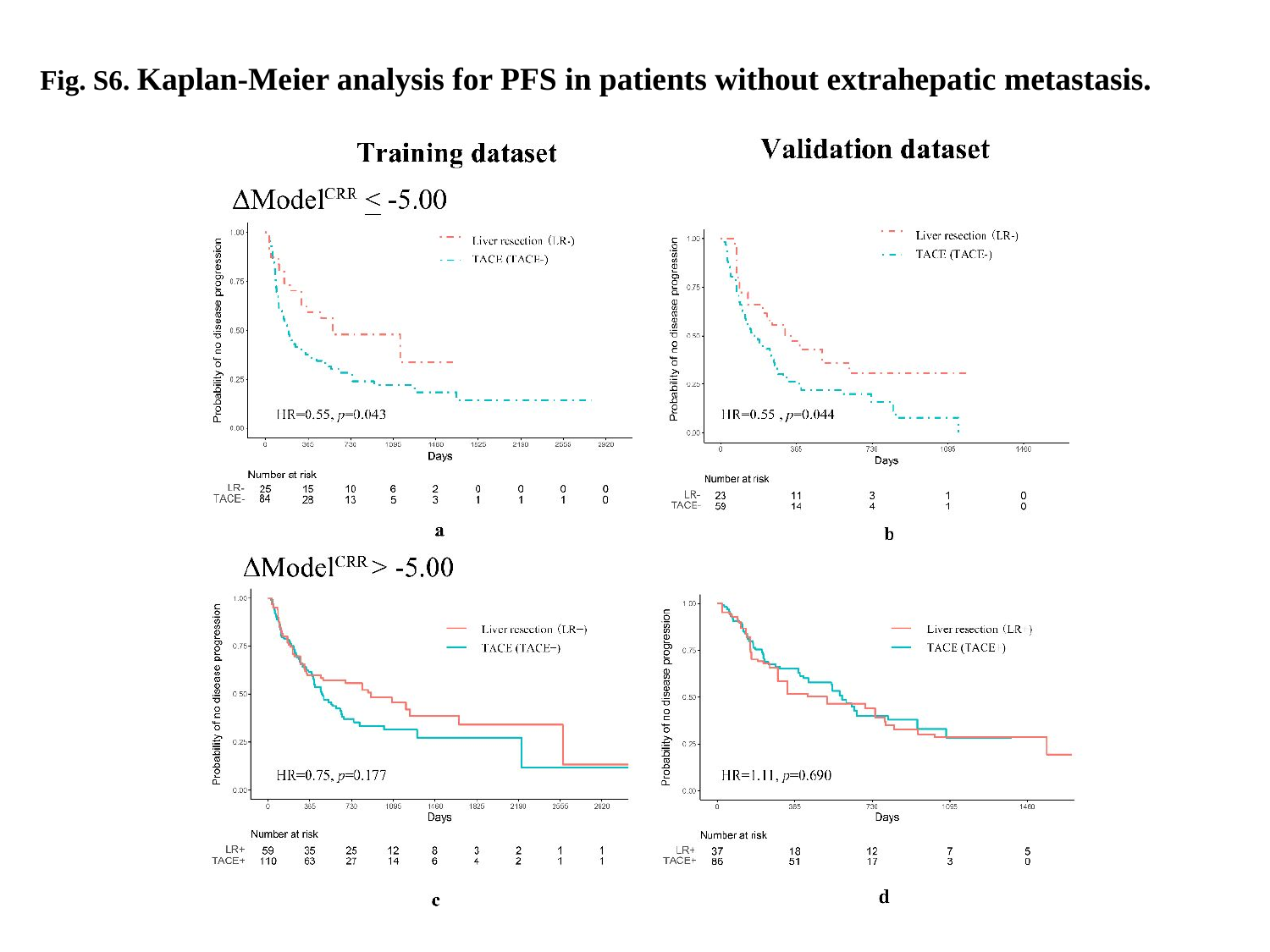

Fig. S6. Kaplan-Meier analysis for PFS in patients without extrahepatic metastasis.
